# Effect of (Tb+Y)/Al ratio on Microstructure Evolution and Densification Process of (Tb_0.6_Y_0.4_)_3_Al_5_O_12_ Transparent Ceramics

**DOI:** 10.3390/ma12020300

**Published:** 2019-01-18

**Authors:** Zhong Wan, Yinzhen Wang, Jian Zhang, Shiwei Wang, Dan Han, Junping Wang, Dewen Wang

**Affiliations:** 1 Guangdong Engineering Technology Research Center of Efficient Green Energy and Environmental Protection Materials, Guangdong Key Laboratory of Quantum Engineering and Quantum Materials, School of Physics & Telecommunication Engineering, South China Normal University, Guangzhou 510006, China; 2016021673@m.scnu.edu.cn; 2 State Key Laboratory of High Performance Ceramics and Superfine Microstructure, Shanghai Institute of Ceramics, Chinese Academy of Sciences, Shanghai 200050, China; swwang51@mail.sic.ac.cn (S.W.); handan@mail.sic.ac.cn (D.H.); wangjunping@mail.sic.ac.cn (J.W.); wangdewen@mail.sic.ac.cn (D.W.); 3 Key Laboratory of Transparent Opto-functional Inorganic Materials, Shanghai Institute of Ceramics, Chinese Academy of Sciences, Shanghai 201899, China; 4 Center of Materials Science and Optoelectronics Engineering, University of Chinese Academy of Sciences, Beijing 100049, China

**Keywords:** (Tb_0.6_Y_0.4_)_3_Al_5_O_12_, densification process, grain growth, HIP treatment

## Abstract

(Tb_0.6_Y_0.4_)_3_Al_5_O_12_ transparent ceramics were successfully fabricated by solid-state reactive sintering using Tb_4_O_7_, Y_2_O_3_, and α-Al_2_O_3_ powders as raw materials. The effect of (Tb+Y)/Al ratio on microstructure evolution and densification process was investigated in detailed. The results showed that the grain growth kinetics were significantly affected by (Tb+Y)/Al ratio. Al-rich and Tb-rich phases appeared in part of the samples of different ratios. Particularly, excess aluminum increased the diffusing process, leading to a higher densification rate, while samples with excess terbium ratios displayed a smaller grain size and lower relative density. The optical quality was highly related to the amount of the secondary phase produced by different (Tb+Y)/Al ratios. Finally, (Tb_0.6_Y_0.4_)_3_Al_5_O_12_ transparent ceramics have been fabricated through pre-sintering in vacuum, followed by hot isostatic sintering (HIP), and the best transmittance of sample with a 4 mm thickness was approximately 78% at 1064 nm.

## 1. Introduction

Magneto-optical material, including glass, single crystal, and transparent ceramic, is the crucial constitution of the optical isolators in high-power laser systems [1,2,3]. At present, due to the advantages of large Verdet constant, high thermal conductive, and low absorption, Tb_3_Ga_5_O_12_ (TGG) is one of the most commonly used commercial magneto-optical material of Faraday isolators [4,5,6]. Compared to TGG, Tb_3_Al_5_O_12_ (TAG) has a higher Verdet constant, which makes it a highly sought magneto-optical isolator material for future applications [7]. However, it is difficult to obtain TAG single crystals, due to incongruent melting [8,9,10]. Although many efforts have been devoted to solving this problem, the size of crystals is still too limited to meet the requirement of practical application [11,12]. This phenomenon can be effectively avoided by fabricating TAG transparent ceramic below the phase transition point, thanks to the cubic structure. 

TAG transparent ceramic has been studied for many years since it was firstly reported in 2011 [13]. A large number of studies have been done investigating the preparation method, ion doping, and magneto-optical property improvements. More importantly, it was found that Y-doping can avoid strain generation and crack initiation during the sintering process. Chen et al. [14] successfully fabricated (Tb_1−x_R_x_)_3_Al_5_O_12_ (R = Y, Ce) ceramics by a two-step sintering method, and confirmed that Y^3+^ addition improved the optical quality of the TAG ceramics. Duan et al. [15] found Y-doping can optimize the microstructure of the TAG transparent ceramics and achieve a smaller average grain size. In 2017, Ikesue et al. [16] produced (Tb_1−x_Y_x_)_3_Al_5_O_12_ transparent ceramics with ultralow optical loss for practical applications, promoting the commercial development of TAG transparent ceramics.

Even though most former studies have claimed that highly transparent TAG or TAG-based ceramics were fabricated, there was still numerous scatters that existed in the samples (pores, second phases, impurities, grain boundaries) which limited further improvement of the transmittance. Generally, in order to avoid the second phase, the ratio of RE/Al (RE is rare earth, such as Y, Lu, Dy) must be carefully controlled as 3/5, according to the binary phase diagram of RE_2_O_3_-Al_2_O_3_. Much research has been devoted to understanding the effect of composition deviation on several common garnet structures. Hu et al. [17] found that excess lutetium restrained abnormal grain growth by the impurity drag effect, while excess Al_2_O_3_ pinned in the grain boundary limited the fast migration of grain boundaries in Pr: LuAG transparent ceramics. Stanek et al. [18] studied the variation of lattice parameter with stoichiometry deviation, and non-stoichiometry in YAG proceeded through cation antisite defects, which would be a theoretical foundation in vacancy diffusion during the densification process. Liu et al. [19] investigated that a small excess of yttrium was tolerable for the optical quality of ceramics compared with excess alumina. They deduced that the average grain size abruptly decreased, and the porosity increased with the increasing of both excess Al_2_O_3_ and Y_2_O_3_. However, related works have not been carried out in TAG ceramic system, though it would be meaningful for obtaining transparent ceramics with excellent magneto-optical properties.

Generally, Tb_4_O_7_ instead of Tb_2_O_3_ is usually used as the raw material to prepare TAG transparent ceramics, owning to the instability of Tb_2_O_3_ at room temperature [20,21]. However, the precise contents of Tb^3+^ and Tb^4+^ in Tb_4_O_7_ powder can be hardly measured. In other words, Tb_4_O_7_ should actually be described as Tb_4_O_7±x_, making precise control of Tb/Al ratios impossible. Therefore, investigate different ratios will be significant for fabrication of high optical quality ceramics. In this paper, (Tb_0.6_Y_0.4_)_3_Al_5_O_12_ transparent ceramics were fabricated by reactive sintering in vacuum, followed by hot isostatic sintering (HIP) treatment and Tb_4_O_7_, Y_2_O_3_, and α-Al_2_O_3_ were used as raw powders. The effect of (Tb+Y)/Al ratio on the phase formation, densification process, and microstructure evolution was elaborately investigated.

## 2. Materials and Methods 

### 2.1. Experimental Procedure

(Tb_0.6_Y_0.4_)_3_Al_5_O_12_ transparent ceramics with different (Tb+Y)/Al mole ratios (0.5964, 0.6000, 0.6036, 0.6073, and 0.6110) were fabricated with high-purity commercial Tb_4_O_7_ (99.99%, Jiahua Corp. Ltd., Jiangyin, China), Y_2_O_3_ (99.99%, Jiahua Corp. Ltd., Jiangyin, China), and α-Al_2_O_3_ (99.99%, Sumitomo Chemicals, Tokyo, Japan) powders. The particle sizes of three raw powders were 2, 1, and 0.2 μm, respectively. Meanwhile, 0.5 wt % TEOS (99.99%, Alfa Aesar Company, Beijing, China) and 0.1 wt % MgO (99.99%, Alfa Aesar Company, Beijing, China) were added as sintering aids. The powder mixtures was dispersed in 99.99% ethyl alcohol and ball-milled in nylon tank for 15 h. Then, the slurries were dried at 80 °C in oven for 24 h and sieved through a 100-mesh screen. After this, they were uniaxially pressed into plates in Φ 12 mm stainless steel molds at 20 MPa and cold isostatic pressing at 200 MPa for 5 min. In order to remove organics, plates were calcined at 800 °C for 6 h in a muffle furnace. The green bodies were pre-sintered at varieties temperatures (from 950 to 1550 °C) in a vacuum furnace (ZW-50-20, Chenrong Corp. Ltd., Shanghai, China) under a vacuum level of ~10^−3^ Pa for 4 h, followed by HIP at 1600 °C under 196 MPa Ar atmosphere. Finally, all samples were annealed at 1350 °C for 10 h in a muffle furnace (SSX-2-16, Yifeng Corp. Ltd., Shanghai, China), and mirror-polished to 4 mm on double sides.

### 2.2. Characterization

The phase compositions of pre-sintering plates were identified by X-ray diffraction (XRD; D2, Bruker, Hamburg, Germany) with Cu Kα radiation. The microstructures of the ceramics were characterized by scanning electron microscopy (SEM; JSM-6510, JEOL, Akishima, Japan). The element mapping was conducted with energy dispersive spectroscopy (EDS; SwiftED3000, HITACHI, Tokyo, Japan). The densities of the transparent ceramics were measured by Archimedes method. The in-line transmittances of the polished samples were measured by UV–vis–NIR spectrophotometer (Lambda 950; Perkin-Elmer, Waltham, MA, USA).

## 3. Results and Discussion

### 3.1. Phase Formation Process

**Figure 1:**
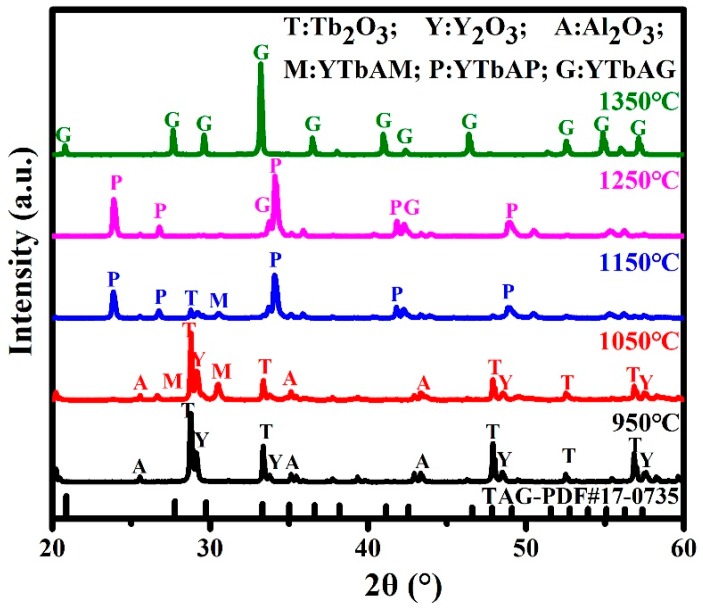
XRD patterns of (Tb_0.6_Y_0.4_)_3_Al_5_O_12_ pre-sintered every 100 °C from 950 to 1350 °C, in vacuum.

The X-ray diffraction patterns in Figure 1 demonstrate the phase formation of the pre-sintered samples with 0.6000 ratio. The results confirm that Tb and Y react with Al_2_O_3_ and form a solid solution of yttrium terbium aluminum garnet (YTbAG). Specifically, Tb_4_O_7_ deoxygenates to Tb_2_O_3_ beyond 950 °C and Tb_2_O_3_, Y_2_O_3_, and Al_2_O_3_ can be detected at this temperature. Yttrium terbium aluminum monoclinic phase (YTbAM) forms at 1050 °C while the diffractions of raw powders still exist. With the temperature increasing, YTbAM and yttrium terbium aluminum perovskite phase (YTbAP) simultaneously appear at 1150 °C, and the diffraction intensity of YTbAM decreases. Meanwhile, YTbAP and some YTbAG are detected at 1250 °C while YTbAM has disappeared. When the temperature reaches 1350 °C, a pure YTbAG phase is generated and all peaks match well with TAG standard card (PDF#17-0735). No residual intermediate phases remain to be detected. In summary, YTbAM, YTbAP, and YTbAG appear in order with the reaction processing, which can be described by the formulas

Tb_4_O_7_ → Tb_2_O_3_ + O_2_ (beyond 950 °C), (1)

Tb_2_O_3_ + Y_2_O_3_ + Al_2_O_3_ → YTbAM (950–1050 °C), (2)

YTbAM + Al_2_O_3_ → YTbAP (1050–1250 °C), (3)

YTbAP + Al_2_O_3_ → YTbAG (1250–1350 °C). (4)

### 3.2. Densification and Microstructure

**Figure 2:**
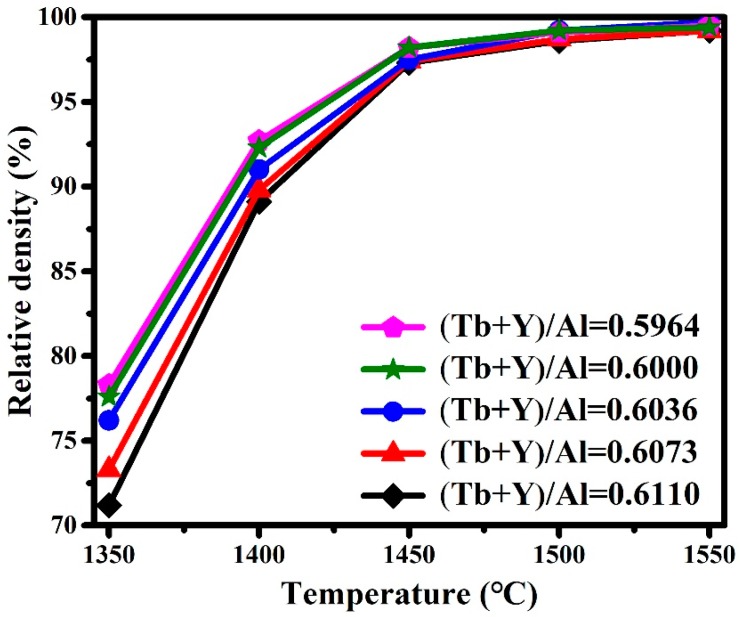
Relationship between relative density and pre-sintering temperature with different (Tb+Y)/Al ratios.

The relationship between relative density and pre-sintering temperature is shown in Figure 2. This indicated that the relative densities improved simultaneously with the increase of temperature for all samples. A rapid densification process between 1350 and 1450 °C can be observed, and the rate slows down from 1450 to 1550 °C. The density fluctuations tend to be flat when the temperature continues to increase. Regularly, the relative density decreases with the ratio of (Tb+Y)/Al increasing. The relative density of the 0.5964 ratio sample is 78%, while it is just 71% for the 0.6110 ratio sample at 1350 °C. Density distinction among different ratio samples constantly decreases as the pre-sintering temperature rises further. Finally, all of the densities with the different (Tb+Y)/Al ratios are almost coincident at 1550 °C, and they are above 99%.

**Figure 3:**
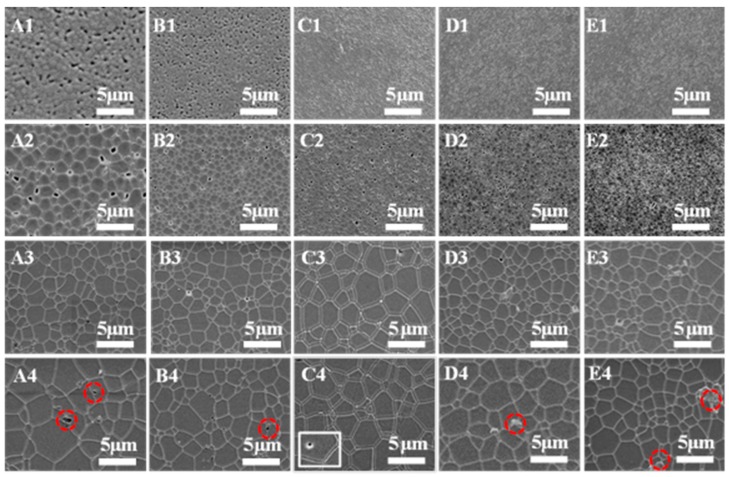
SEM micrographs of the thermal etching surface of different (Tb+Y)/Al ratio samples (**A**–**E** represent 0.5964, 0.6000, 0.6036, 0.6073 and 0.6110) pre-sintered at different temperatures (1 to 4 represent 1350, 1400, 1450, and 1500 °C).

Figure 3 shows the thermal etching surface of the as-prepared ceramics with different (Tb+Y)/Al ratios (0.5964, 0.6000, 0.6036, 0.6073, and 0.6110) pre-sintered from 1350 to 1500 °C. It is clearly observed that the average grain size of ceramics increases and the porosity decreases with the sintering temperature increasing, regardless of (Tb+Y)/Al ratios. Open pores can be easily observed at 1350 °C which change to being closed around 1400 °C. When the sintering temperature reaches 1450 °C, the samples possess uniform grains as well as high density. The microstructure evolution as well as porosity changes are consistent with the results displayed in Figure 2. It is worth mentioning that second phases at grain boundaries appear in part of the (Tb+Y)/Al ratio samples (0.5964, 0.6000, 0.6073, and 0.6110) when the sintering temperature reaches 1450 °C, and they are marked with red circles. For samples with (Tb+Y)/Al ratios of 0.5964 and 0.6000, the residual pores and average grain size are obviously larger than those samples with other ratios (0.6073 and 0.6110) below 1450 °C. The density distinction decreases with continually increasing temperature. However, for the sample with (Tb+Y)/Al ratio of 0.6036, the grain boundaries are clean and free from second phases and abnormal grains at each temperature. Unluckily, intergranular pores appear at 1500 °C and are unable to be removed by HIP treatment. 

**Figure 4:**
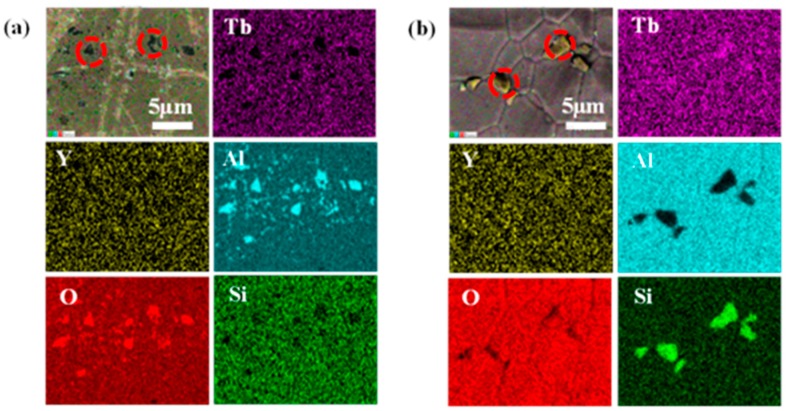
Energy dispersive spectroscopy of two ratios pre-sintered at 1450 °C: (**a**) 0.6000, (**b**) 0.6073.

SEM micrograph and the corresponding EDS mapping images of various elements of the pre-sintered sample with two (Tb+Y)/Al ratios (0.6000 and 0.6073) are shown in Figure 4. Figure 4a indicates that Al_2_O_3_ second phases are detected in the sample with a ratio of 0.6000. Due to the lower atomic number, it looks darker. Meanwhile, Tb and Y disappear in this area. The mapping result of 0.6073 ratio is showed in Figure 4b, and excess Tb second phases exist in the bright area. Surprisingly, an interesting phenomenon occurs that the content of Si is also enriched. This may be caused by generation of a terbium silicate compound. Similar results were already reported in Nd: YAG transparent ceramics [22,23].

The rare earth-controlled densification and average grain size were already discovered in YAG [24,25]. In this investigation, it can be explained that (Tb+Y)/Al ratios affect the sintering behavior via generating structural defects which can promote or inhibit densification and grain growth, relying on their atoms’ diffusion kinetics. When excess Al reacts with terbium in the system, it generates a high concentration of vacancies, which increases the diffusion rate during the sintering process. The second phase does not produce a pinning effect, and the grain boundaries migrate, sequentially, with higher temperature. When Tb is in excess, it consumes the bulk concentration of rare earth vacancies so that the diffusion kinetics of rare earth species would be limited. This also indicates that excess terbium hinders the grain growth which is significantly effective at lower temperatures.

**Figure 5:**
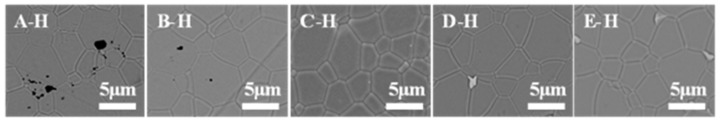
SEM images of different (Tb+Y)/Al ratio samples (**A**–**E** represent 0.5964, 0.6000, 0.6036, 0.6073 and 0.6110) pre-sintered at 1450 °C and with 1600 °C HIP treatment.

HIP treatment is a typical method in fabricating optical ceramics, because it can remove residual intergranular pores and further improve the densification. There is more suitable microstructure of pre-sintering ceramics before HIP due to no intragranular pores, higher relative density, and smaller grain size. Therefore, the microstructures of samples pre-sintered at 1450 °C and treated by 1600 °C HIP are displayed in Figure 5. It can be seen that residual pores are removed through HIP treatment. At the same time, the average grain size also obviously grows despite the second phases remain in some samples. 

### 3.3. Optical Quality

**Figure 6:**
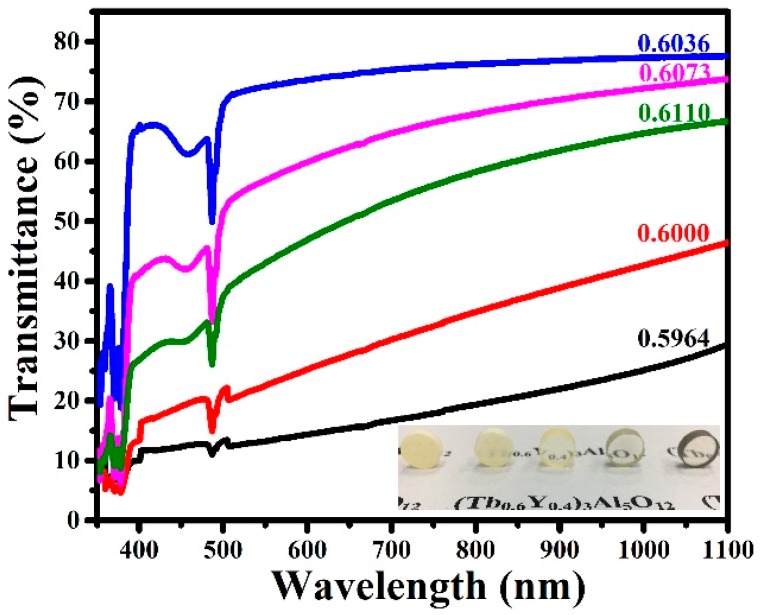
Transmittances of different (Tb+Y)/Al ratios pre-sintered at 1450 °C and with 1600 °C HIP treatment (4 mm thickness); Macroscopic observations of (Tb_0.6_Y_0.4_)_3_Al_5_O_12_ with different (Tb+Y)/Al ratios are displayed inside: from left to right (0.5964, 0.6000, 0.6036, 0.6073, and 0.6110).

Figure 6 shows the in-line transmittance curves of samples pre-sintered at 1450 °C followed by 1600 °C HIP treatment. Specifically, excess aluminum enormously affects the transmittance both in the visible and near-infrared region, while the transmittance lines of excess terbium samples are lower than the sample with 0.6036 ratio, and it decreases quickly along with the wavelength decreasing. Actually, this phenomenon is explained by the Mie scattering caused by residual pores [26]. The absorption peak at 484 nm is attributed to the Tb^3+^: ^7^F_6_ → ^5^D_4_ transition. The best optical quality sample, whose transmittance reaches 78% at 1064 nm, is obtained by a (Tb+Y)/Al ratio of 0.6036. Macroscopic observations of (Tb_0.6_Y_0.4_)_3_Al_5_O_12_ transparent ceramics with different (Tb+Y)/Al ratios are displayed inside. The samples with 0.6036, 0.6073, and 0.6110 ratios are transparent, with words being clearly seen below the photograph, while 0.5964 and 0.6000 ratio samples are opaque. The bright yellow appearance is connected with the valence state of terbium.

## 4. Conclusions

(Tb_0.6_Y_0.4_)_3_Al_5_O_12_ transparent ceramics were fabricated by vacuum pre-sintering and HIP treatment. Due to the uncertain volume of terbium in Tb_4_O_7_ raw powders, the influence of different (Tb+Y)/Al ratios on the densification process and optical properties of (Tb_0.6_Y_0.4_)_3_Al_5_O_12_ transparent ceramics were studied in detail. Meanwhile, results indicated that excess aluminum ((Tb+Y)/Al = 0.5964 and 0.6000) made the densification process faster, while excess terbium ((Tb+Y)/Al = 0.6073 and 0.6110) caused decay and hindered grain growth. More importantly, the excess aluminum or terbium caused second phases to appear, which seriously affected the sample optical properties. Finally, transparent ceramic with (Tb+Y)/Al = 0.6036 pre-sintered at 1450 °C in vacuum, followed by HIP treatment at 1600 °C, resulted in better optical quality and a transmittance of up to 78% at 1064 nm in a sample of 4 mm thickness was obtained.
